# Influence of cytokine inhibitors on concentration and activity of MMP-1 and MMP-3 in disc herniation

**DOI:** 10.1186/ar2858

**Published:** 2009-11-11

**Authors:** Stéphane Genevay, Axel Finckh, Françoise Mezin, Enrico Tessitore, Pierre-André Guerne

**Affiliations:** 1 Division of Rheumatology, University Hospitals of Geneva, Avenue Beau-Séjour 26, 1211 Geneva 14, Switzerland; 2 Division of Neurosurgery, University Hospitals of Geneva, rue Gabrielle-Perret-Gentil 4, 1211 Geneva 14, Switzerland

## Abstract

**Introduction:**

Spontaneous resorption of disc herniation (DH) after sciatica is well documented. The matrix metalloproteinases (MMP)-1 and MMP-3 are enzymes potentially involved in this process. Glucocorticoid injections are commonly used for treatment, and other anti-inflammatory molecules like tumor necrosis factor (TNF) inhibitors are under clinical investigation. However, little is known about the effect of these molecules on DH resorption.

**Methods:**

DH tissue was harvested from patients undergoing surgery for sciatica. Samples were thoroughly washed. Diced explants were cultured ex-vivo in 1) 0.5 ml Dulbecco's modified Eagle's medium (DMEM) 10% fetal calf serum (FCS), (controls), 2) recombinant interleukin 1 receptor antagonist (IL-1Ra), (100 ng/ml), 3) dexamethasone (10E-5 M), or 4) TNF inhibitor monoclonal antibody (10 μg/ml). Supernatants were harvested at 48 hours and frozen. Immunocapture activity assays determined total MMP activity, active MMP levels and pro-MMP levels.

**Results:**

Fourteen DH tissue samples were analysed. Levels of all forms of MMP-3 were higher than the respective levels of MMP-1(*P* < 0.01). In particular, the median (interquartile range [IQR]) total MMP-3 level was 0.97 (0.47 - 2.19) ng/mg of tissue compared to 0.024 (0.01 - 0.07) ng/mg of total MMP-1 level (*P* < 0.01). Incubation with IL-1Ra, dexamethasone, or TNF inhibitors significantly decreased levels of all forms of MMP-3 (*P* < 0.05). Dexamethasone significantly decreased the ratio of active MMP-3 to total MMP-3 activity. A significant inhibitory effect of dexamethasone was observed only on active MMP-1, while IL-1 and TNF inhibitor had no significant effect on any form.

**Conclusions:**

MMP-3 appears to play a greater role than MMP-1 in DH resorption. Dexamethasone, IL-1-Ra and TNF inhibitor decreased active MMP-3, indicating that the clinical use of these drugs may affect the resorption of DH under certain conditions.

## Introduction

Disc herniation (DH) is classically described as the protrusion of degenerated disc tissue within the spinal canal [1]. Although DH is found in many asymptomatic subjects, lumbar DH is associated with radicular leg pain syndrome often referred to as sciatica. While sciatica was long thought to result only from mechanical compression of the nerve root, recent studies have underlined the importance of inflammation and cytokines in this process. Partly for this reason, glucocorticoids [2] and, more recently, TNFα inhibitors [3,4] were introduced in the treatment of sciatica. The usual clinical evolution of sciatica is toward recovery with resolution of leg pain. Reduction in clinical symptoms has been shown to be correlated with a reduction of DH size on subsequent magnetic resonance imaging [5].

Matrix metalloproteinases (MMPs) are a group of over 20 zinc-dependent enzymes that catalyze the degradation of protein components of the extracellular matrix. MMPs therefore contribute to the tissue resorption and remodeling of the extracellular matrix that occur in reaction to tissue degeneration [6]. MMP-1 (collagease-1) and MMP-3 (stromelysin-1) are known to be involved in the turnover of normal tissue but also in its pathological degradation. Osteoarthritis [7,8], spondyloarthropathy [9] or intervertebral disc (IVD) degeneration [10] illustrates this process. MMPs have also been shown to be increased in DH tissue compared with that of healthy IVDs [11] and participate in DH degradation and resorption after an episode of sciatica [12]. Little information is available, however, on their respective importance in this process.

Synthesized as inactive pro-zymogens, MMPs go through a post-transcriptional process of cleavage and activation, enabling the targeted degradation of their substrate. The regulation of MMP activity is a complex and finely tuned process in which both specific inhibitors (tissue inhibitors of metalloproteinases) and the regulation of afferent pathways at production and activation levels play an important part. Inflammatory cytokines such as IL-1 and TNFα are thought to contribute to these regulatory processes [7]. The use of glucocorticoids [2] and TNF inhibitors [3,4] in the treatment of sciatica might therefore hinder DH resorption and, possibly, the median or long-term evolution of the disease.

The goal of the present study was therefore to investigate the effects of glucocortiocoids (dexamethasone) and specific cytokine inhibitors (IL-1Ra and anti-TNF antibody) on levels of MMP-1 and MMP-3 in DH. We used assays that distinguish active enzymes from inactive enzymes to partially address the level of regulation at which these drugs might be active.

## Materials and methods

The local research ethics committee's approval was given for the work. DH tissues were obtained after informed consent from 14 patients undergoing surgical lumbar discectomy for persistent radicular symptoms. No patients had received glucocortiocoids within 2 weeks prior to surgery and none had received IL-1 or TNF inhibitors at any time.

Freshly obtained tissue samples were immediately transported in a dry environment to the laboratory, thoroughly washed with DMEM in order to remove any blood contamination, and diced into pieces of approximately 50 mg. The time duration between sample collection and processing did not exceed 1 hour. Histological analysis was performed on the first two DH samples. All samples were subsequently incubated *ex vivo *at 37°C for 48 hours either in 0.5 ml DMEM supplemented with 10% FCS, in penicillin and streptomycin alone (controls), or with the addition of dexamethasone (10^-5 ^M), IL-1 Ra (100 ng/ml; R&D Systems Europe Ltd, Abingdon, Oxfordshire, UK) or anti-TNFα monoclonal antibodies (10 ng/ml; R&D Systems Europe Ltd). Concentrations of anti-inflammatory molecules were chosen to match those reached in tissue *in vivo *after systemic administration. All tissue samples were incubated in duplicate. At the end of the incubation period, supernatants were harvested, aliquoted and stored at -80°C until assays were performed.

### Immunocapture activity assays

**Figure 1:**
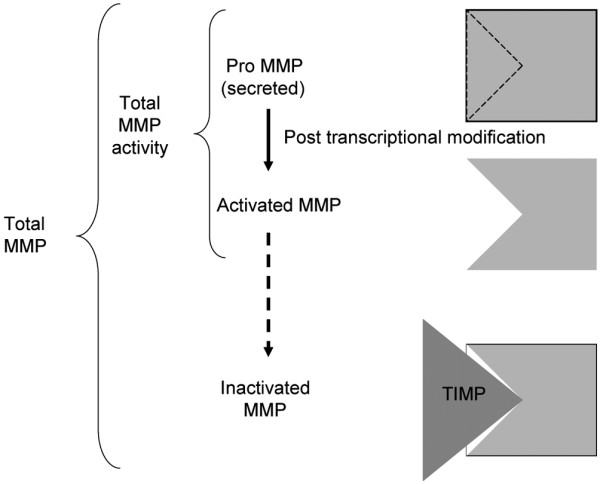
Different forms of matrix metalloproteinase enzymes and their usual nomenclature. Immunocapture activity assays (used in the present study) detect both total matrix metalloproteinase (MMP) activity and active MMP, while the standard ELISA method detects total MMP (inactive, active and inactivated forms). TIMP, tissue inhibitor of matrix metalloproteinase.

MMP-1 and MMP-3 activity assays (Amersham Biosciences, Little Chalfont, Buckinghamshire, UK) were performed according to the recommendations of the supplier. These assays measure spontaneous MMP activity (active MMP) and total MMP activity after activation (total MMP activity = spontaneously active + activatable pro-MMP; Figure 1), but do not capture MMP-proteinase inhibitor complexes (inactivated MMP). Total MMP, frequently referring to the sum of all three forms (pro-MMP, active MMP and inactivated MMP) because the usual immunoassays capture indistinctly all three forms at the same time, was not measured in the present study.

In the activity assay used in this study, MMP-1 or MMP-3 is captured by a specific antibody that has been immobilized on a microtiter plate. The amount of active MMP is measured directly by the incubation of the captured MMP with modified pro-urokinase (Ukcol, Amersham Biosciences), and subsequently activated Ukcol is quantified by a chromogenic substrate (S-2444, Amersham Biosciences). Color development is recorded at 405 nm at different time intervals. Total MMP activity (in the present study, the pro-MMP plus active MMP) is assessed through the activation of pro-MMP by preincubation with 0.5 mmol/l *p*-aminophenylmercuric acetate for 2 hours at 37°C before the addition of modified Ukcol and chromogenic substrate.

The level of pro-MMP can be computed by subtracting active MMP from total MMP activity (Figure 1). Activity is expressed in recombinant enzyme equivalents in nanograms per milliliter. The lower detection limits were 1.7 ng/ml recombinant enzyme equivalents for MMP-1 and 3.75 ng/ml recombinant enzyme equivalents for MMP-3. Results were adjusted for the dry weight of each sample and are presented as nanograms per milligrams (ng/mg) of tissue.

### Statistical analysis

As results were not normally distributed, they are presented as median (interquartile range (IQR)), displaying the first (25%) and the third (75%) quartiles. A nonparametric Wilcoxon signed rank sum test was used to compare the levels of each MMP after incubation with, respectively, IL-1Ra, dexamethasone, or TNF inhibitor with the levels of MMPs measured in the control samples. All tests were conducted at an alpha level of error of 0.05. Calculations were performed with STATA v. 9.2 for Windows (StataCorp LP, College Station TX, USA).

## Results

DH tissue was collected during surgery from 14 patients (Table 1) operated for DH and sciatica unresponsive to conventional conservative treatment. Most patients had a history of chronic back pain prior to the acute episode of leg pain; two patients had already suffered from a previous episode of radiculopathy. None of them had had prior back surgery. Leg pain duration ranged from 3 to 200 weeks at the time of surgery. All patients had a positive straight leg raise sign or radicular irritation sign and/or a definite neurological deficit. Seven of them had a combination of two or three neurological signs. According to visual examination by the surgeons during surgery, 11 DH samples were subligamentous and three DH samples had crossed the posterior ligament.

**Table 1:** Baseline characteristics of the patients

	Disc herniation samples
Sample size	14
Age (years)	45 (15)
Gender (% male)	45
Leg pain duration (weeks)	14 (10 to 40)
Type of back pain	
Acute	5
Chronic	10
Type of leg pain	
First episode	13
Previous episodes	2
Positive nerve root irritation sign^a^	14
Neurological deficit	
Muscle weakness	8
Sensory perturbation in a dermatome	9
Decrease deep tendon reflex	5
Disc level	
L3 to L4	2
L4 to L5	7
L5 to S1	6

Data presented as *n*, mean (standard deviation) or median (interquartile range). ^a^Straight leg-raising or femoral stretch.

### Basal matrix metalloproteinase levels

Levels of active MMP-1 and total MMP-1 activity were analyzed in 13 DH samples. The median (IQR) level of total MMP-1 activity was 0.024 (0.01 to 0.07) ng/mg tissue and was 0.006 (0 to 0.03) ng/mg tissue for active MMP-1. The median (IQR) level of pro-MMP-1 was 0.01 (0.06 to 0.04) ng/mg tissue.

Levels of active and total MMP-3 activity were analyzed in 13 DH samples, 11 of them being the same tissue as that used for MMP-1 level determination. The median (IQR) level for total MMP-3 activity was 0.97 (0.47 to 2.19) ng/mg tissue and was 0.25 (0.17 to 0.75) ng/mg tissue for active MMP-3. The median (IQR) level of pro-MMP-3 was 0.47 (0.19 to 1.34) ng/mg tissue.

**Figure 2:**
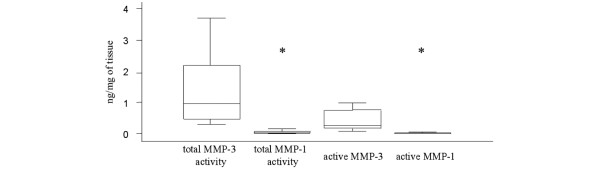
Levels of matrix metalloproteinase-3 and matrix metalloproteinase-1. Total matrix metalloproteinase (MMP)-3 activity and active MMP-3 levels were found at significantly higher levels than their respective MMP-1 form, *P < 0.01. Line in center of box, median; lower and upper borders of boxes, lower (Q1) and upper (Q3) quartiles; whiskers, minimum and maximum values; o, outliers (values exceeding 1.5 × (Q3 - Q1)).

The median levels of total MMP-3 activity, active MMP-3 and pro-MMP-3 were found to be more than 30 times higher than those of the respective forms of MMP-1, *P *< 0.01 (Figure 2). None of the clinical characteristics as displayed in Table 1, such as duration of leg pain before surgery or the extension of DH beyond the posterior ligament, were significantly correlated with any of the MMP-1 or MMP-3 levels.

### Effect of inhibitors on matrix metalloproteinases

**Figure 3:**
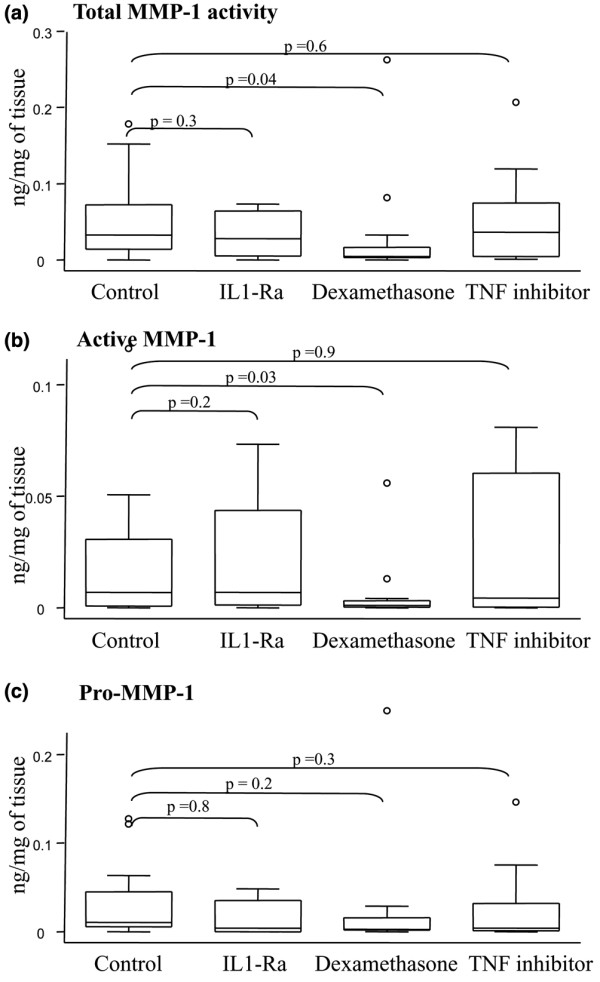
Effect of IL-1Ra, dexamethasone and TNF inhibitor on matrix metalloproteinase-1. Dexamethasone significantly inhibits both **(a) **total matrix metalloproteinase (MMP)-1 activity and **(b) **active MMP-1 without modifying levels of **(c) **pro-MMP-1. No effect was observed for IL-1Ra and TNF inhibitor. Line in center box, median; lower and upper borders of boxes, lower (Q1) and upper (Q3) quartiles; whiskers, minimum and maximum values; o, outliers (values exceeding 1.5 × (Q3 - Q1)).

Incubation with dexamethasone significantly decreased the concentrations of total MMP-1 activity from 0.03 (0.01 to 0.07) ng/mg tissue to 0.004 (0.003 to 0.017) ng/mg tissue, *P *= 0.04, and of active MMP-1 from 0.007 (0.0007 to 0.03) ng/mg tissue to 0.001 (0.0003 to 0.003) ng/mg tissue, *P *= 0.03. Dexamethasone did not, however, affect the concentration of pro-MMP-1. Incubation with IL-1Ra, which could be analyzed in only eight samples, or with TNF inhibitor did not affect the concentration of any forms of MMP-1 (Figure 3). Addition of the anti-inflammatory drugs did not significantly modify the ratio of active MMP-1 to total MMP-1 activity.

**Figure 4:**
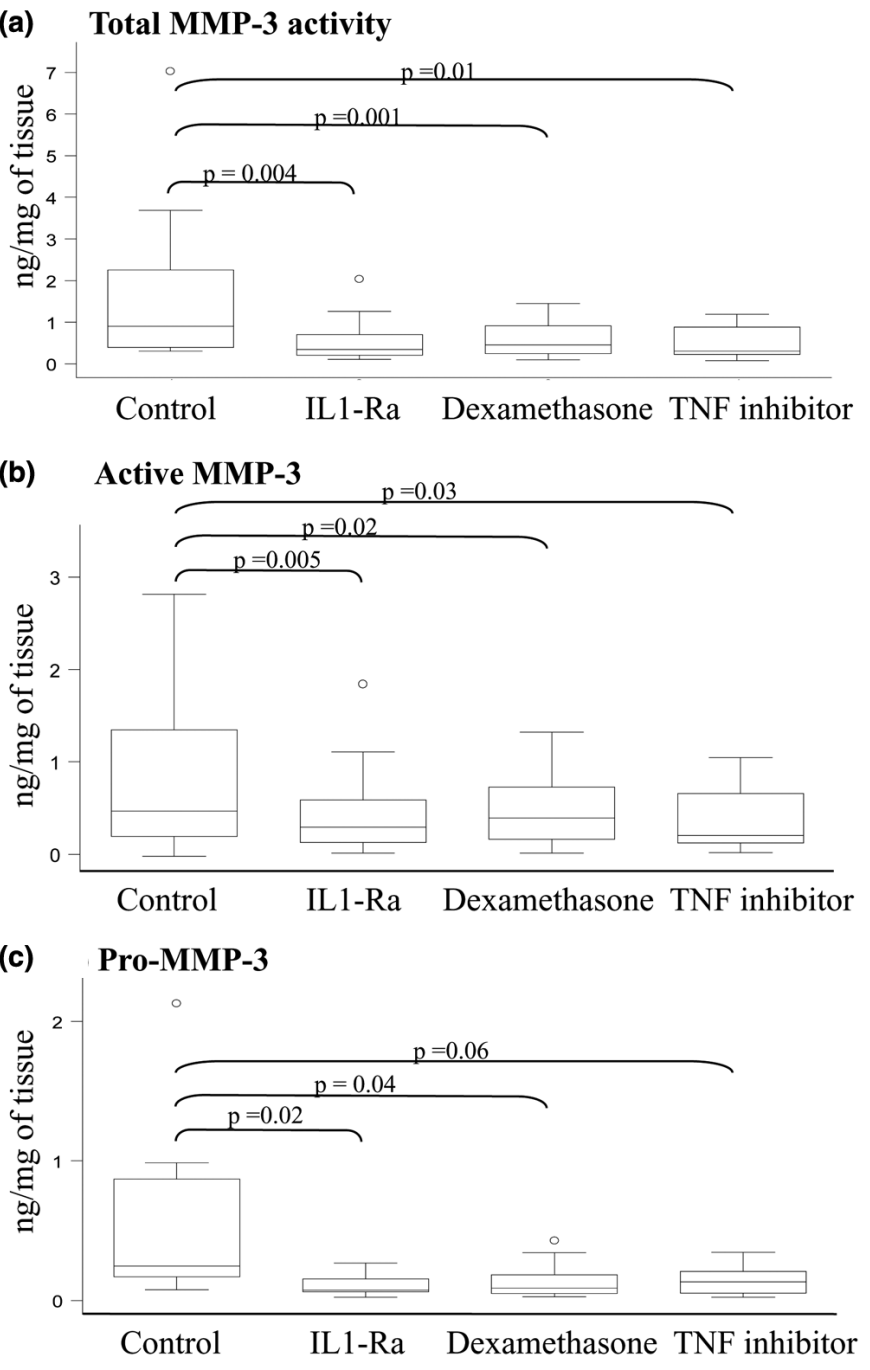
Effect of IL-1Ra, dexamethasone and TNF inhibitor on matrix metalloproteinase-3. All three inhibitors decreased levels of **(a) **total matrix metalloproteinase (MMP)-3 activity and **(b) **active MMP-3 without modifying levels of **(c) **pro-MMP-3. Line in center of box, median; lower and upper borders of boxes, lower (Q1) and upper (Q3) quartiles; whiskers, minimum and maximum values; o, outliers (values exceeding 1.5 × (Q3 - Q1)).

Incubation with dexamethasone significantly decreased the concentrations of all forms of MMP-3. The total MMP-3 activity level went down from 0.9 (0.4 to 2.26) ng/mg tissue to 0.46 (0.25 to 0.91) ng/mg tissue, *P *= 0.01, that of active MMP-3 from 0.25 (0.17 to 0.87) ng/mg tissue to 0.09 (0.05 to 0.19) ng/mg tissue, *P *= 0.02, and that of computed pro-MMP-3 from 0.47 (0.19 to 1.34) ng/mg tissue to 0.39 (0.16 to 0.72) ng/mg tissue, *P *= 0.04 (Figure 4).

Incubation with IL-1Ra significantly decreased the concentrations of all forms of MMP-3. The total MMP-3 activity level went down to 0.35 (0.21 to 0.7) ng/mg tissue, *P *< 0.01, that of active MMP-3 to 0.07 (0.06 to 0.15) ng/mg tissue, *P *< 0.01, and that of computed pro-MMP-3 to 0.29 (0.13 to 0.59) ng/mg tissue, *P *= 0.02 (Figure 4).

Incubation with TNF inhibitor significantly decreased the concentration of total MMP-3 activity to 0.3 (0.22 to 0.88) ng/mg tissue, *P *= 0.01, and that of active MMP-3 to 0.13 (0.53 to 0.21) ng/mg tissue, *P *= 0.03. There was a nonsignificant decrease in the level of pro-MMP-3 to 0.2 (0.12 to 0.66) ng/mg tissue, *P *= 0.06 (Figure 4).

The addition of dexamethasone decreased the ratio of active MMP-3 to Total MMP-3 activity from 39% (25.6 to 53.9%) to 20.1% (16.0 to 25.0%), *P *= 0.02, whereas no significant difference was observed with IL-1Ra and TNF inhibitor.

## Discussion

In the present study, all measured forms of MMP-3 were found at higher concentrations than the corresponding forms of MMP-1, suggesting that MMP-3 plays a greater role in DH degradation than MMP-1. Our results inform on the process of DH resorption, given the known specificities of these MMPs. MMP-1 alone degrades native intact interstitial collagen molecules. MMP-3 mainly contributes to the degradation of many noncollagenous proteins, but it also breaks down previously denatured collagen fibrils [10]. The presence of both enzymes in DH tissue was previously reported [13], as was the effect of MMP-3 on DH resorption [14]. These studies, however, did not provide any information on the respective importance of MMP-1 and MMP-3 in this process. In IVD tissue, MMP-1 is expressed at a higher level than MMP-3 [12,15]. The proportion of MMP-1 decreases with advancing disc degeneration, and both enzymes are equally expressed in the most degenerative IVD [15]. Our results suggest that DH tissue resorption may be even more dependent on MMP-3 than end-stage degenerative IVD tissue. Further comparison with our study is difficult as no IVD studies reported any information on MMP levels or activity.

Each of the three drugs studied had a different effect on MMP-1 and MMP-3 concentrations. IL-1Ra and TNF inhibitor only affected MMP-3 levels, whereas dexamethasone was effective in decreasing concentrations of both MMP-1 and MMP-3. To the best of our knowledge, this study is the first to report a direct effect (that is, without prior *in vitro *cell activation) of either IL-1Ra, TNF inhibitors or dexamethasone on the levels of MMP-1 and MMP-3 in DH tissue. Moreover, the anti-inflammatory agents' impact was observed in active components of both MMP-1 and MMP-3, suggesting that using these drugs could, at least under certain conditions, alter the speed and extent of DH resorption after an episode of sciatica. Such an effect was not reported after one foraminal injection of methylprednisolone in patients with sciatica [16], but was observed in a rabbit model using higher dosages [17]. Regarding TNF inhibitors, a single infusion of infliximab [18] showed no effect on DH resorption at 6-month follow-up, although resorption was observed to slow down during the first 2 weeks post infusion, compared with patients having received a placebo. This time lag coincides with the 10 days serum half-life of the drug, suggesting that repetitive infusion might affect disc resorption.

IL-1 Ra, but not TNF inhibitors, was recently reported to inhibit matrix degradation in IVD [19]. This result differs both from our findings and from a report of decreased serum levels of MMP-3 in patients with spondyloarthropathy treated with TNF inhibitors [20]. A difference in tissue extracellular matrixcomposition between healthy IVD and DH tissue might account for this discrepancy. Alternatively, the immunocapture activity assay that was used in the present study specifically addresses each form of the enzyme, whereas the zymography used for the study on IVD assesses a global effect on substrate and precludes any conclusions for a specific enzyme [21]. As for dexamethasone, no data on DH or IVD tissue are available. The inhibitory effect on MMP-3 levels, however, has been reported in chondrocytes [22] and in the serum level in rheumatoid arthritis patients after intra-articular injection of glucocorticoids [23].

Many mechanisms are involved in the regulation of active MMP levels: gene activation and repression, post-translational acetylation, DNA methylation, post-transcriptional modification of pro-MMP with complex activation of zymogens by free radicals and other enzymes, and inhibition of active MMPs by numerous molecules, including α_2_-macroglobulin and tissue inhibitors of metalloproteinases. The present study was not designed to investigate these different mechanisms. The immunocapture activity assay, however, enabled the assessment of the different forms of MMPs and permits some preliminary observations. The inhibitory effect of dexamethasone on active MMP-1, without modifying the ratio between active MMP-1 and total MMP-1 activity, rather suggests an increase in inhibitory molecules. Indeed, such an effect of dexamethasone has been reported on tissue inhibitor of metalloproteinase-1 [24]. The impact of dexamethasone on pro-MMP-3 suggests an upstream effect on the pathways implicated in the synthesis of this enzyme. Because of the additional effect observed on the ratio of active MMP-3 to total MMP-3 activity, however, a more complex effect on pathways of MMP-3 regulation may be postulated. Further work is certainly needed to clarify these aspects.

The present study presents other limitations. First, the complex process of extracellular matrix regulation involves other, possibly yet unknown, enzymes in addition to MMP-1 and MMP-3. As DH tissue has not been studied in as much detail as degenerative IVD tissue, we chose to focus on what are thought to be two major MMPs, to the exclusion of less well-known molecules. Now that the results of the present study suggest that the metabolism of DH tissue is somewhat different from that of IVD tissue, further research is needed.

Second, for technical reasons, DH tissues had to be incubated for 48 hours, in contrast to *in vivo *conditions, where the effects of dexamethasone and cytokine inhibitors might be quantitatively different. We do not, however, expect this to affect qualitatively our conclusions.

The third limitation is that the *in vitro *conditions and the added inhibitors might also have affected cell viability. The active synthesis of MMPs *in vitro *speaks against large cell death, however, and the concentrations of anti-inflammatory molecules were chosen to match those reached in tissues *in vivo *after systemic administration.

The duration of symptoms prior to surgery varied widely and could have lead to differences in the DH environment. Nevertheless, statistical analysis showed that duration of symptoms was not correlated with any of the MMPs levels.

Finally, magnetic resonance imaging studies have demonstrated that the speed and degree of DH resorption after an episode of sciatica are related to the extension of DH in relation to the posterior longitudinal ligament, with transligamentous DH tending to shrink faster. This may be due to the quantity of granulation tissue present around the DH. In the present study, unfortunately, only three samples of transligamentous DH were available, which precludes any definitive conclusion on this topic.

## Conclusions

MMP-3 is present at higher levels than MMP-1 in DH, suggesting it plays a predominant role in DH resorption. The effect of dexamethasone and TNF inhibitors on the active forms of MMP3 suggests that, at least under certain circumstances, the clinical use of these drugs may affect the resorption of DH. These results indicate in particular that further research on the medium and long-term evolution of DH size and resorption after TNF inhibitor treatment or glucocorticoid treatment is warranted.

## Abbreviations

DH: disc herniation; DMEM: Dulbecco's modified Eagle's medium; ELISA: enzyme-linked immunosorbent assay; FCS: fetal calf serum; IL: interleukin; IQR: interquartile range; IVD: intervertebral disc; MMP: matrix metalloproteinase; TNF: tumor necrosis factor.

## Competing interests

The authors declare that they have no competing interests.

## Authors' contributions

SG and P-AG were responsible for the study design, interpretation of the data and manuscript preparation. AF carried out the statistical analysis. ET was responsible for sample tissue collection. FM and P-AG were responsible for immunocapture activity assay. All authors reviewed and approved the final manuscript.
